# Prostate cancer arising in ectopic prostatic tissue within the left seminal vesicle: a rare case diagnosed with multi-parametric magnetic resonance imaging and magnetic resonance imaging-transrectal ultrasound fusion biopsy

**DOI:** 10.1186/s12880-016-0122-3

**Published:** 2016-02-24

**Authors:** Alexander S. Somwaru, Deepu Alex, Atif K. Zaheer

**Affiliations:** Department of Radiology, CCC Building, MedStar Georgetown University Hospital, 3800 Reservoir Road, N.W., Washington, DC 20007 USA; Department of Pathology and Laboratory Medicine, MedStar Georgetown University Hospital, Washington, DC USA; Russell H. Morgan Department of Radiology and Radiological Science, Johns Hopkins Medical Institutions, Baltimore, MD USA

## Abstract

**Background:**

Benign ectopia of prostatic glandular tissue in the seminal vesicles is rare with only three prior cases reported in the literature. Prostate cancer, arising within prostatic ectopia in the seminal vesicles, has never been described and therefore presents a challenge in both diagnosis and management.

**Case presentation:**

Herein, we report a rare case of prostatic adenocarcinoma in ectopic prostate tissue in the left seminal vesicle, without evidence of prostatic glandular involvement. This case was diagnosed on multi-parametric magnetic resonance imaging and confirmed with magnetic resonance imaging-transrectal ultrasound fusion biopsy.

**Conclusions:**

Awareness of this unusual phenomenon is significant because of the potential occurrence of malignancy to arise in unexpected, extra-glandular locations, which are not routinely sampled on routine transrectal ultrasound biopsy. However attention to lesions with characteristic multi-parametric magnetic resonance imaging features of prostate cancer, regardless of extra-glandular location, will help direct tissue sampling, facilitate a timely diagnosis and ensure appropriate management.

## Background

An 80 year-old male, with a past medical history of benign prostatic hyperplasia (BPH), presented to Urology with a rising serum prostate specific antigen (PSA) level of 13.1 ng/mL. In 2008 and again in 2012, the patient underwent two 12-core TRUS biopsies, which yielded negative results. Given his rising PSA level, the patient subsequently underwent diagnostic multi-parametric prostate MR imaging to localize any potentially malignant lesions that may have been missed on biopsies.

## Case presentation

**Fig. 1:**
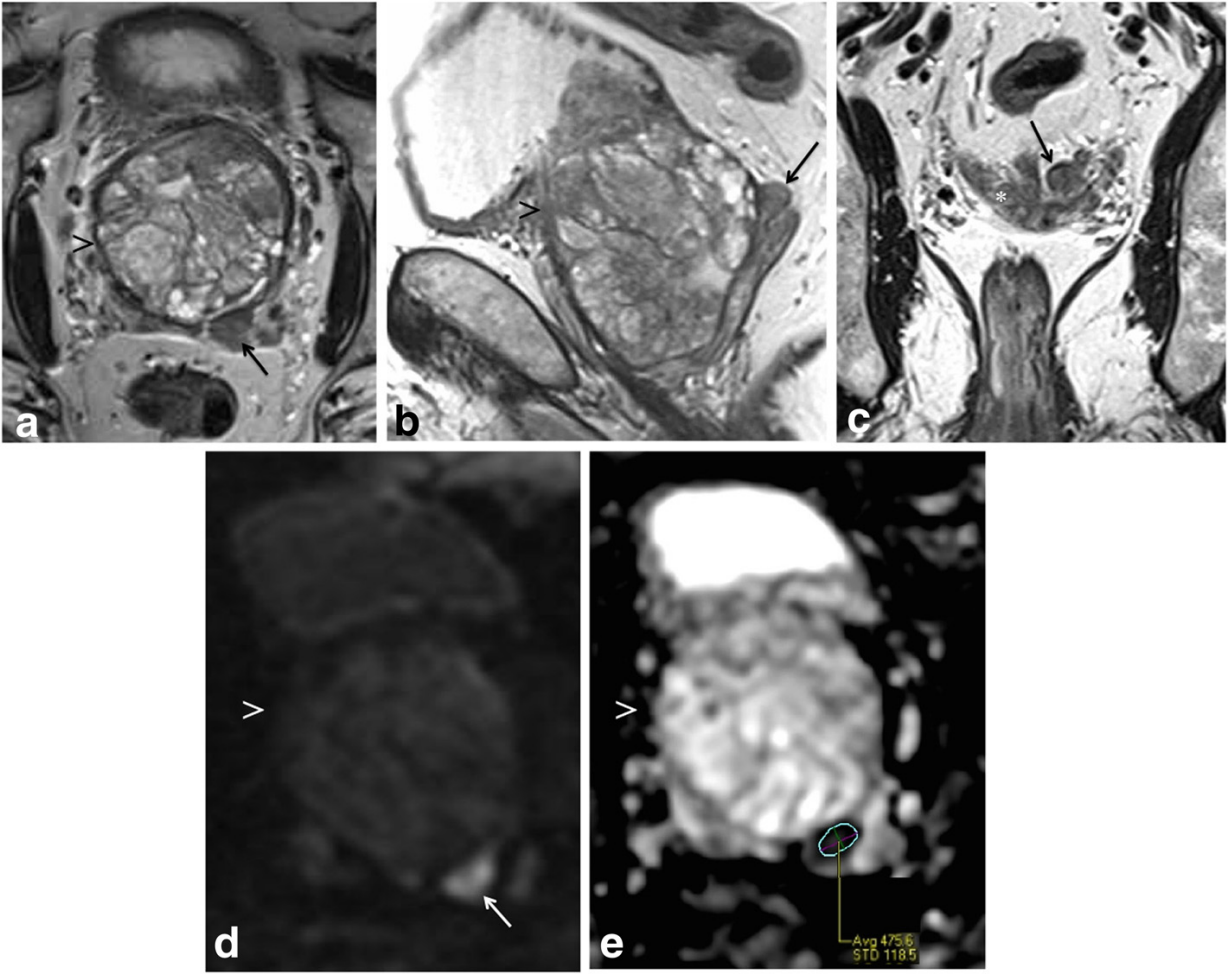
Multi-parametric magnetic resonance (MR) imaging of the prostate gland demonstrates normal prostate gland however an abnormal lesion in the left seminal vesicle features classical imaging features of prostate carcinoma. **a** High-resolution axial T2-weighted (T2W) image demonstrates an abnormal 1.5 × 1.2 × 1.0 cm lesion with relative hypointensity (*arrow*) subjacent to an enlarged prostate gland (*arrowhead*). The capsule is maintained without trans-capsular extension. **b**–**c** High-resolution multi-planar T2W images in the sagittal and coronal planes show, to advantage, this lesion within the left seminal vesicle (*arrow*), external to the capsule of the prostate gland (*arrowhead*). No T2W signal intensity abnormality is in the adjacent left prostate gland base peripheral or transitional zone. Moreover, there is no exophytic hyperplastic nodule or contiguous extension of prostatic glandular tissue related to the abnormality in the left seminal vesicle. Normal right seminal vesicle denoted for comparison (*asterisk*). **d** High *b*-value diffusion weighted imaging (DWI) shows that this lesion demonstrates significantly diminished diffusivity (*arrow*). **e** Apparent diffusion coefficient (ADC) map confirms significantly diminished diffusivity in this lesion (*arrow*) with low ADC mean value of 0.5 × 10^−3^ mm^2^/s (*region of interest*). No diminished diffusivity was seen in the prostate gland (*arrowhead*)

**Fig. 2:**
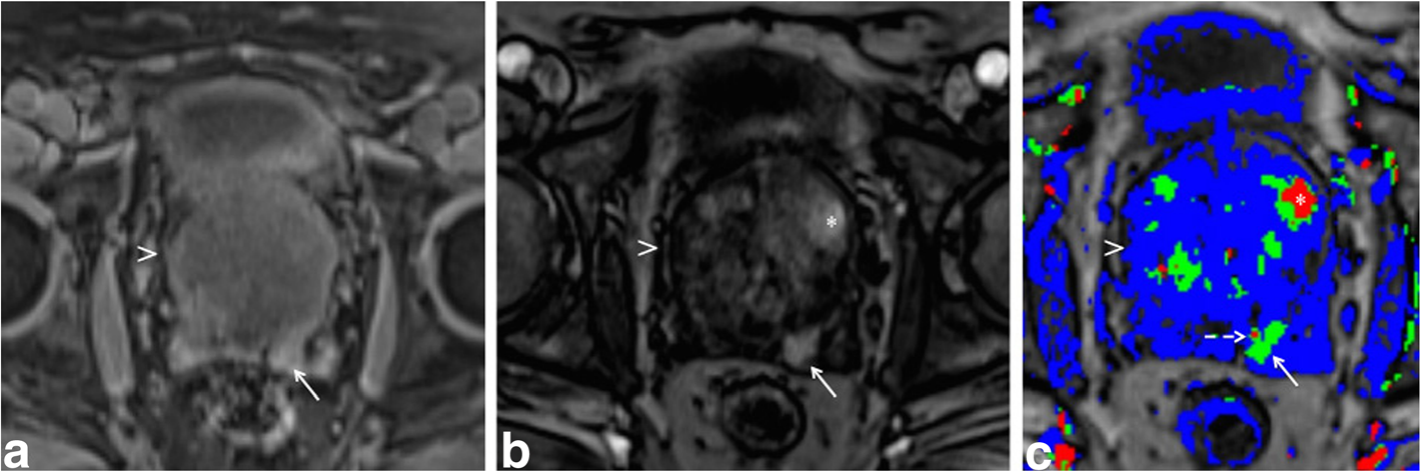
**a**-**b** Pre- and post-contrast fat-saturated T1-weighted (T1W) imaging of the prostate gland (*arrowhead*) and left seminal vesicle (*arrow*) reveal that this lesion demonstrated abnormal perfusion on dynamic contrast enhancement (DCE). **c** Computer-aided detection (CAD) confirms abnormal perfusion pattern of the lesion in the left seminal vesicle, which demonstrates early enhancement with partial washout and colorimetric stratification of both indeterminate/suspicious (‘*green*’) (*dashed arrow*) and highly suspicious patterns (‘*red’*) (*solid arrow*). The hyperperfused lesion in the left central zone with highly suspicious enhancement on CAD (*asterisk*) demonstrated relative hypointensity on T2W imaging with thin, circumscribed hypointense periphery, and no diminished diffusivity, compatible with a hyperplastic (BPH) stromal nodule that was later confirmed as benign on MR imaging-TRUS fusion biopsy

**Fig. 3:**
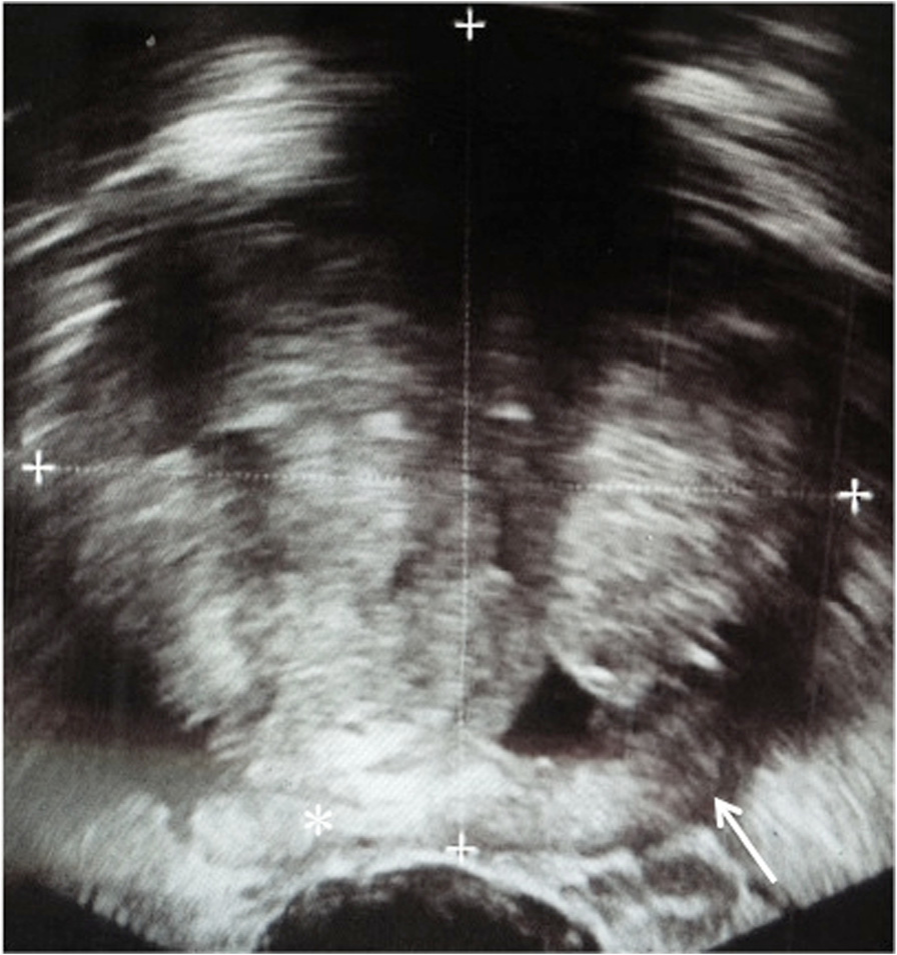
Gray-scale ultrasound image from MR imaging-TRUS fusion biopsy demonstrates the abnormal, hypoechoic lesion in the left seminal vesicle (*arrow*); normal right seminal vesicle for comparison (*asterisk*) and prostate gland (*cross-hairs*)

Multi-parametric prostate MR imaging revealed no abnormality in the central, transitional or peripheral zones of the prostate gland. However, in the left seminal vesicle and non-contiguous with the adjacent prostate gland or capsule, there was an abnormal 1.5 × 1.2 × 1.0 cm lesion with conspicuous hypointense T2-weighted (T2W) signal intensity seen on high-resolution axial imaging (Fig. 1a). High-resolution coronal and sagittal T2W images show, to advantage, this lesion within the left seminal vesicle (Fig. 1b-c), external to the prostate gland capsule, which is maintained with normal signal intensity and morphology. No T2W signal intensity abnormality is seen in the adjacent left prostate gland base related to the left seminal vesicular abnormality. Moreover, there is no exophytic hyperplastic nodule or contiguous extension of prostatic glandular tissue related to the abnormality in the left seminal vesicle. On high *b*-value diffusion weighted imaging (DWI) and apparent diffusion coefficient (ADC) map, this lesion features significantly diminished diffusivity with low ADC mean value of 0.5 × 10^−3^ mm^2^/s (Fig. 1d-e). There is no abnormal diffusivity in the adjacent or regional left prostate gland base peripheral or transitional zone related to the left seminal vesicular abnormality. Pre- and post-contrast fat-saturated T1-weighted (T1W) imaging obtained prior to and following the infusion of intravenous gadolinium contrast show abnormal perfusion of this lesion (Fig. 2a-b). Abnormal perfusion pattern of this lesion is confirmed with CAD, which demonstrates early enhancement with partial washout and colorimetric stratification of ‘green’ and ‘red’: indeterminate/suspicious (‘green’) and highly suspicious (‘red’) (Fig. 2c dashed arrow and solid arrow). The hyperperfused lesion in the left central zone with highly suspicious enhancement (Fig. 2c asterisk) demonstrated relative hypointensity on T2W imaging with thin, circumscribed hypointense periphery, and no diminished diffusivity, compatible with a hyperplastic (BPH) stromal nodule that was later confirmed on MR imaging-TRUS fusion biopsy with no malignancy on tissue histopathological sampling. This lesion was reported as clinically significant cancer is highly likely to be present; it was subsequently targeted on MR imaging-TRUS software-based fusion biopsy. Repeat 14-core TRUS biopsy, supplemented with targeted MR imaging-ultrasound fusion-guidance, was performed of the prostate gland and left seminal vesicle. Ultrasound gray-scale imaging from the fusion biopsy confirms an abnormal hypoechoic lesion in the left seminal vesicle (Fig. 3).

Tissue histology from the repeat 14-core biopsies revealed benign prostatic tissue in all prostate gland sextants (12 cores), particularly in the left prostate base (3 total cores). However core biopsies of the lesion in the left seminal vesicle revealed both malignant and benign prostatic tissue.

**Fig. 4:**
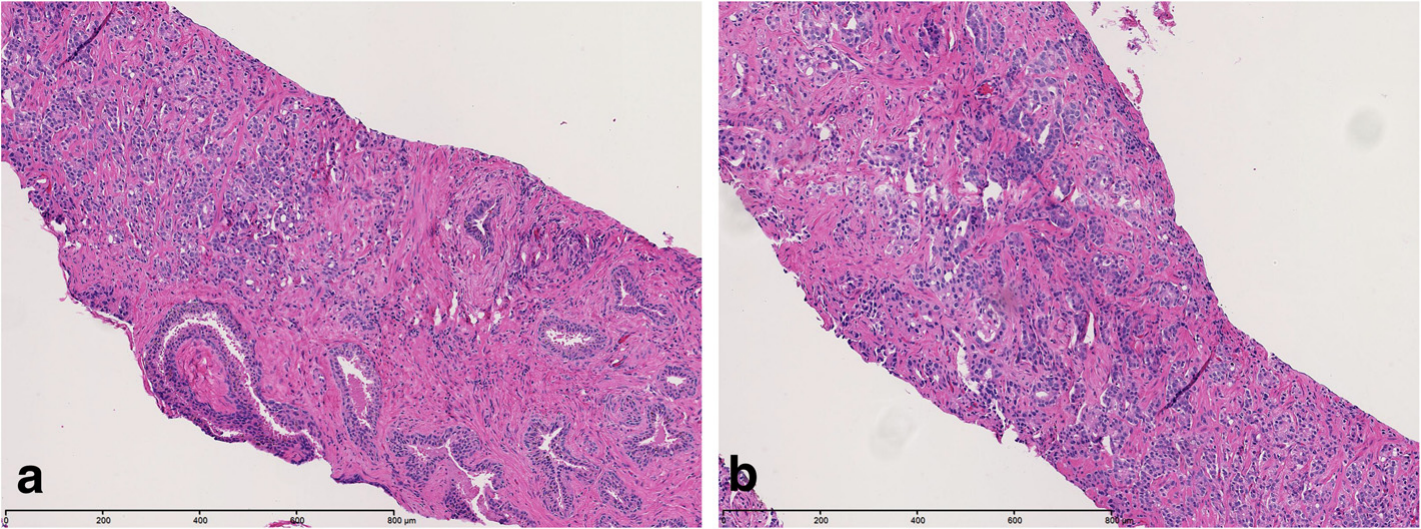
**a** Photomicrographs from tissue histopathological examination reveals prostate adenocarcinoma, Gleason 4 pattern (*left of image*), characterized by collections of neoplastic cells with minimal gland formation, with benign prostatic glandular elements and seminal vesicular tissue (*right of image*), H&E stain image, 10× magnification. **b** Prostate adenocarcinoma, predominant Gleason 4 pattern and secondary Gleason 3 pattern, H&E stain image, 10× magnification. The carcinoma involves approximately 60 % of the submitted core biopsy fragments

**Fig. 5:**
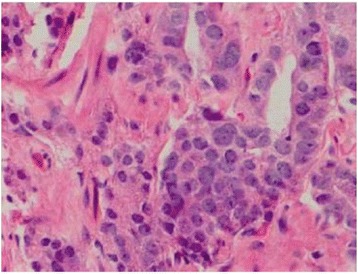
Prostate adenocarcinoma, predominant Gleason 4 pattern and secondary Gleason 3 pattern (Gleason 4 + 3 = 7), H&E stain image, 400× magnification

Fusion-guided targeted core biopsy of the left seminal vesicular lesion demonstrated prostate adenocarcinoma with a predominant Gleason four pattern, characterized by collections of neoplastic cells with minimal gland formation, surrounded by benign prostatic glandular elements within benign seminal vesicular tissue (Fig. 4a). A secondary Gleason three pattern is identified with recognizable neoplastic glands (Fig. 4b). The carcinoma involves approximately 60 % of the submitted core biopsy fragments (Fig. 5). The presence of benign prostatic glandular elements in seminal vesicular tissues is abnormal, confirming the presence of prostatic ectopia. Moreover, alpha-methylacyl-CoA racemase over-expression confirms the presence of prostate carcinoma. Multiple fusion-guided targeted biopsies of the adjacent left prostate gland base peripheral and transition zones revealed only benign prostatic tissue with no malignancy. Following complete diagnostic workup, staging, and multi-disciplinary discussion of therapeutic options with the Departments of Urology, Radiation Oncology, and Pathology, the patient was treated as stage III disease (T3bN0M0) and underwent external beam radiation therapy (EBRT) and hormonal therapy.

While the phenomenon of ectopic prostatic glandular tissue in the genitourinary tract is rare, it is not entirely uncommon. While the majority of cases of benign prostatic ectopia have been reported in the urinary bladder [1, 2] and urethra [3], cases have also been described in the retro-vesicular space [4, 5], seminal vesicles [6–8], epididymis [9], testis [10], and rectum [11]. Cancer arising in prostatic ectopia has only been reported four times in the literature, all in the urinary bladder and urethra [12–15]. However we report a case of primary prostate adenocarcinoma arising in the seminal vesicle, without glandular or trans-capsular involvement, which to our knowledge, has never been described.

## Conclusions

Due to its infrequency, malignancy arising within prostatic ectopia in the seminal vesicles has never been described and therefore presents a challenge in both diagnosis and management. Awareness of this unusual phenomenon is significant because of the potential occurrence of malignancy to arise in unexpected, extra-glandular locations, which are not routinely sampled on routine transrectal ultrasound biopsy. However attention to lesions with characteristic multi-parametric magnetic resonance imaging features of prostate cancer, regardless of extra-glandular location, will help direct tissue sampling, facilitate a timely diagnosis and ensure appropriate management.

## Consent

Written informed consent was obtained from the patient for publication of this case report and any accompanying images. A copy of the written consent is available for review by the Editor-in-Chief of this journal.

## Abbreviations

ADC: apparent diffusion coefficient
BPH: benign prostatic hyperplasia
CAD: computer-aided detection
DWI: diffusion weighted-imaging
EBRT: external beam radiation therapy
ECE: extra-capsular extension
MR: magnetic resonance
PSA: prostate-specific antigen
T1W: T1-wieghted
T2W: T2-weighted
TRUS: transrectal ultrasound
