# Erratum to: prostate cancer arising in ectopic prostatic tissue within the left seminal vesicle: a rare case diagnosed with multi-parametric magnetic resonance imaging and magnetic resonance imaging-transrectal ultrasound fusion biopsy

**DOI:** 10.1186/s12880-016-0132-1

**Published:** 2016-04-26

**Authors:** Alexander S. Somwaru, Deepu Alex, Atif Zaheer

**Affiliations:** Department of Radiology, CCC Building, MedStar Georgetown University Hospital, 3800 Reservoir Road, N.W., Washington, DC 20007 USA; Department of Pathology and Laboratory Medicine, MedStar Georgetown University Hospital, Washington, DC USA; Russell H. Morgan Department of Radiology and Radiological Science, Johns Hopkins Medical Institutions, Baltimore, MD USA

## Erratum

Unfortunately, the original version of this article [1] contained an error. The spelling of the author “Atif Zaheer” was misspelt “Atif K. Zaheer”. This has been corrected in the original article and can also be seen correctly in the author list above.
